# Supplementary material for: Barriers and facilitators to informal healthcare provider engagement in the national tuberculosis elimination program of India: An exploratory study from West Bengal
Source: PLOS Glob Public Health. 2023 Oct 4;3(10):e0001390. doi: 10.1371/journal.pgph.0001390 (PMC10550149; doi:10.1371/journal.pgph.0001390)
Supplement: S1 File — (PDF) [file pgph.0001390.s001.pdf]

Rapid review of literature:

Title: Rapid review of studies on engaging/involving/integrate/collaborating informal healthcare providers (IPs)

Search Strategy:

Key words 1: “Perspective*” OR “Opinion*” OR “Perception*” OR “View*” OR “Understand*”

AND

Key words 2: “Integrat*” OR “Engage*” OR “Involve*” OR “Participat*”

AND

Key words 3: 64 key terms for Informal Healthcare Providers

AND

Key words 4: “Qualitative research” OR “Qualitative stud*” OR “Qualitative method*” OR “Qualitative interview*”

Note: The search was not limited to tuberculosis for two reasons.

- Studies in tuberculosis care are limited.
- Keeping it broader will provide an opportunity to understand the overall exiting literature on this topic.

Inclusion criteria:

- Qualitative studies
- IPs are defined broadly as a care provider working outside the purview of formal health system and lack qualification for the kind of services they offer. It also includes CHWs who are not part of the formal health system.
- Studies focusing on provider and/or stakeholders and/or patients’ perspective on some form of integration, involvement, or engagement.
- English language

Exclusion criteria:

- Studies focused on patients’ perspectives regarding use of healthcare, illness, service provision (with IPs) without a component on integration, involvement, or engagement.
- Studies focused on integration of human resources, not just health services.
- Quantitative studies

EMBASE search:

S. N	Terms	Count
1	Keywords 1	2956633
2	Keywords 2	4027967
3	Keywords 3	106971
4	Keywords 4	128429
5	1 AND 2 AND 3 AND 4	775

MEDLINE search:

S. N	Terms	Count
1	Keywords 1	2,400,934
2	Keywords 2	3,277,117
3	Keywords 3	40687
4	Keywords 4	110,770
5	1 AND 2 AND 3 AND 4	745

CINAHL search:

S. N	Terms	Count
1	Keywords 1	656,928
2	Keywords 2	645,708
3	Keywords 3	27,829
4	Keywords 4	139,213
5	1 AND 2 AND 3 AND 4	347

Title and abstract screening: 1286 (Done by one person)

Full-text review: 36 (Done by one person)

Final included: 13

Review findings summary:

Table 1: Included studies

S. N	Author (Year)	Study title
**1**	Pinkoane (2008)	Policy makers' perceptions and attitudes regarding incorporation of traditional healers into the national health care delivery system
**2**	Sieverding (2016)	Integrating informal providers into a people-centered health systems approach: qualitative evidence from local health systems in rural Nigeria
**3**	Nemutandan (2016)	Perceptions and experiences of allopathic health practitioners on collaboration with traditional health practitioners in post-apartheid South Africa
**4**	Hindley (2016)	The role of traditional and faith healers in the treatment of dementia in Tanzania and the potential for collaboration with allopathic healthcare services
5	Musyimi (2017	Integration of Traditional Birth Attendants into Mental Healthcare: A Multistakeholder Qualitative Study Exploration
**6**	Gupta (2017)	Can community health workers play a greater role in increasing access to medical abortion services? A qualitative study
**7**	Orya (2017)	Strengthening close to community provision of maternal health services in fragile settings: an exploration of the changing roles of TBAs in Sierra Leone and Somaliland
**8**	Wilunda (2017)	Changing the role of traditional birth attendants in Yirol West County, South Sudan
**9**	Akol (2018)	"We are like co-wives": Traditional healers' views on collaborating with the formal Child and Adolescent Mental Health System in Uganda
**10**	Krah (2018)	Integrating Traditional Healers into the Health Care System: Challenges and Opportunities in Rural Northern Ghana
**11**	Mendu (2019)	Biomedical healthcare and African traditional healing in the management of HIV and AIDS: complimentary or competing cosmologies?
**12**	Deuchar et al (2020)	Establishing views of traditional healers and biomedical practitioners on collaboration in mental health care in Zanzibar: a qualitative pilot study
**13**	Potts (2020)	A Qualitative Evaluation of a Home-based Palliative Care Program Utilizing Community Health Workers in India

Table 2: Study summary (n=13)

S. N	Variables	Frequency
1	Year of publication (in range)	
	2005-2010	1
	2011-2015	0
	2016-2020	12
2	Study region	
	Africa	11
	South Asia	2
3	Provider type*	
	Traditional healers	7
	Traditional birth attendants	3
	CHWs/ Community health volunteers	2
	Medicine vendor	1
	Rural medical practitioners	1
4	Study areas	
	General health system	4
	Maternal health services	3
	Mental health	2
	Child and adolescent health	1
	Dementia	1
	HIV	1
	Palliative care 1	1

We identified an evidence gap in the field of IPs' work in TB care, as most publications focused on areas such as mental health, reproductive health, and general health. In addition, the most common provider types included in these studies were traditional healers and traditional birth attendants. The least featured provider type was untrained allopathic practitioners (UAP) [Table 2].

Table 3: Summary of study methods (n=13)

S. N	Variables	Frequency
1	Data collection method	
	KII	8
	FGD	2
	KII+FGD	3
2	Sampling	
	Purposive	8
	Convenience	3
	Purposive + Convenience	2
3	Approach to data analysis	
	Open coding	4
	Grounded theory	2
	Thematic analysis	4
	Content analysis	2
	Not mentioned	1

Table 4: Various factors identified in the included studies (n=13)

S. N	Author (Year)	Study title	Factors identified
1	Pinkoane (2008)	Policy makers' perceptions and attitudes regarding incorporation of traditional healers into the national health care delivery system	Communication, Policies, Terms and conditions (Guidelines), Recognition and respect.
2	Sieverding (2016)	Integrating informal providers into a people-centered health systems approach: qualitative evidence from local health systems in rural Nigeria	Policies, Desire to help their communities.
3	Nemutandan (2016)	Perceptions and experiences of allopathic health practitioners on collaboration with traditional health practitioners in post-apartheid South Africa	Incompatibility of two health systems, Quality of health care.
4	Hindley (2016)	The role of traditional and faith healers in the treatment of dementia in Tanzania and the potential for collaboration with allopathic healthcare services	Desire to help people, Incentive, Referral link between two system, Knowledge, Loss of business.
5	Musyimi (2017)	Integration of Traditional Birth Attendants into Mental Healthcare: A Multistakeholder Qualitative Study Exploration	Role clarity, TBAs' patient rapport and counselling experience, Recognition and appreciation, Training and collaboration of TBAs with healthcare workers.
6	Gupta (2017)	Can community health workers play a greater role in increasing access to medical abortion services? A qualitative study	Appropriate training, Regular supplies, and job aids.
7	Orya (2017)	Strengthening close to community provision of maternal health services in fragile settings: an exploration of the changing roles of TBAs in Sierra Leone and Somaliland	Appropriate training and support, Embedded and trusted community relationships, Appropriate remuneration of trained TBAs, Strategies to sustain their work.
8	Wilunda (2017)	Changing the role of traditional birth attendants in Yirol West County, South Sudan	Acceptance of the new TBAs' role by the community, women and TBAs, Perceptions about institutional childbirth and risks of home childbirth, Personal commitment and motivation by some TBAs, Good working relationship between community-based TBAs and health facility staff, Availability of incentives for

			women at health facilities, and Training of TBAs, Communication problems between TBAs and health care facilities, Delays in seeking care by women, Insecurity, Lack of materials and supplies for TBAs, Health system constraints, Insufficient incentives for TBAs, Long distances to health facilities and Transportation problems.
9	Akol<br>(2018)	"We are like co-wives": Traditional healers' views on collaborating with the formal Child and Adolescent Mental Health System in Uganda	Traditional healers believe clinicians disregard them, Mutual trust, Doctors despise all our work regard it as satanic and dirty, Competence, Clinical providers would use English language as a means for excluding the less educated traditional healers, Law or policy recognising traditional healers.
10	Krah<br>(2018)	Integrating Traditional Healers into the Health Care System: Challenges and Opportunities in Rural Northern Ghana	Lack of understanding of traditional medicine, Discrimination, High turnover of biomedical staff, Declining interest in healing as a profession, Equipment scarcity, Extensive infrastructure of traditional medicine, Openness to collaboration, Grassroots initiatives.
12	Deuchar et al (2020)	Establishing views of traditional healers and biomedical practitioners on collaboration in mental health care in Zanzibar: a qualitative pilot study	Education of traditional healers in the recognition of mental illness, perceived opportunity to learn additional skills from biomedical practitioners, Clear referral pathways between the two service providers.
13	Potts (2020)	A qualitative evaluation of a home-based palliative care program utilizing community health workers in India	CHWs' desire and need for more training, The need for tailoring of existing intervention protocols and modifying expectations of stakeholders, The need for considerations for ensuring program sustainability.
