# Supplementary material for: Barriers and facilitators to informal healthcare provider engagement in the national tuberculosis elimination program of India: An exploratory study from West Bengal
Source: PLOS Glob Public Health. 2023 Oct 4;3(10):e0001390. doi: 10.1371/journal.pgph.0001390 (PMC10550149; doi:10.1371/journal.pgph.0001390)
Supplement: S2 File — (PDF) [file pgph.0001390.s002.pdf]

For Informal Healthcare Providers:

1. **Please share about your work as an IP in the community?**

   Probes:

- What kind of services do you provide?
- How is your relationship with the community?
- Why do you think people seek care from IPs?

2. **Please share your interaction with the last TB patient.**
3. **Please share your opinion on why IPs are important in the health system?**

   Probes:

- There are government and private health facilities in India, so in this context, are IPs important? If so, why?
- What are the benefits of engaging IPs in the health system?
- Will IPs engagement in the health system benefit community?

4. **What do you think is the level of TB care knowledge among IPs?**

   Probes:

- What can help IPs to improve their knowledge and skills? Ask follow-up questions like; if they mention training, what kind of training, who should provide it? What kind of content needs to be covered?
- Ask them to give an example of the training they felt was good and ask why they felt so.

5. **What kind of support is essential for IPs to work in formal TB program?**

   Probes:

   Support from the government? First, let IPs respond, and later use the following probes to discuss this section.

   Training – Ask what kind of training, duration, who should be the provider, content if any, modality of training

   Incentive or remuneration – More details like how much, monetary, and non-monetary incentives.

   Clear guideline on TB for IPs – What should be in the guideline? Is there any current guideline?

   Support from the government – What kind of support will help IPs to function properly?

   Recognition and appreciation of work? How would IPs feel appreciated for their work? Why is this important for IPs?

6. **Is there any challenge in working with the government in a formal TB program? Please share your opinion.**

   Probes:

- Equal treatment from the formal system – Do you think there might be some discrimination from the formal system?
- Coordination with the formal system – Please explain
- Any other challenge you would like to share – Please explain

7. **What is your opinion; does engagement with the government system affect your work in anyway?**

   Probes:

- Loss of income
- Fear of losing a patient
- Extra work burden
- Reporting challenges

8. **We found that some of the IPs are recently engaged by the government to refer suspected TB patients. Are you also involved in that program? Please share your experience.**

   Probes:

- If yes, how has your experience been so far? Anything that you feel can be improved?
- If not, why are you not participating in this program?
- Do IPs you know all participate in this program? Are they happy with this program?

9. **Have you done any other work with the formal system? Please share your experience.**

   Probes:

- What was your experience? Was there any problem? Would you like to do it again?

10. **Why do you think it is important for the government to engage IPs in TB program? What is your opinion on how IPs engagement will benefit the forma TB program?**
11. **In general, are you happy with how you currently work with the formal system or how the formal system has engaged IPs?**

    Probes:

- If you are happy, why? If not, why do you feel so?
- Do you think IPs are treated equally by formal providers like doctors, ASHAs?
- How can a formal system make engagement with IPs better?

12. **IPs in West Bengal are recognized by the state government. Has it made any difference to your practice?**

    Probes:

- Has it made it easy for IPs to function?
- Has it changed your relationship with the government?
- Do you feel part of the formal system more than before?
- Has it improved your trust in the community?
- Any significant changes after the recognition?
- Any new challenges after the recognition?

13. **If you are invited to work formally in the TB program, would you be willing to work with the government?**

    Probes:

- If yes, why?
- If not, why? What can government do to encourage you to get involved?

14. **What should be the government next step to engage IPs in TB program formally?**

    Probes:

- Provide more clarity on IPs role
- Integrate IPs into the mainstream health system
- Develop guidelines for IPs in TB care
- Provide proper training

Thank you for your participation!!!

For formal providers:

1. **Can you please share about your work in TB care?**

   Probes:

- What is your major/primary role in the TB care program?
- What kind of activities are you involved in the TB care program?

2. **What do you think are the major challenges in the current national TB program? [Based on the area of work you do]**

   Probes:

- Challenges in screening or diagnosing patients?
- Challenges in treatment or follow up?
- Or any other challenges?

Now I will focus our discussion on Informal Providers, who are locally known as RHCPs (Rural healthcare providers) in West Bengal.

3. **Can you describe any experience you have working with an IP in general or in any TB program? Or have you had any chance to interact with IPs in general or any TB related activities?**
4. **In particular to TB, in your opinion, how are IPs providing care to TB patients in the community?**

   Probes:

- Do you think they have the appropriate knowledge to undertake those activities?
- Are they providing quality care to patients?

5. **A significant proportion of TB patients first seek care from IPs, or IPs are the first point of contact. Why do you think such practices exist in the community?**
6. **If IPs are engaged in the TB program, what do you think at what level of the health system can they be involved?**

- Community level
- Periphery health centers level
- District level

7. **Previously you mentioned few gaps (…………………..) in the current national TB care program. Do you think IPs could play a role to narrow down those gaps?**
8. **Why do you think IPs are currently not engaged in TB program formally like other private providers?**

   Probes:

- Policy issues
- Resources (like incentives)
- Unclarity in role
- Trust between formal and informal system
- Quality of care
- IPs' willingness

9. **Do you think engaging IPs could improve the TB care program? If yes, why. If no, why not?**
10. **Are there any benefits of engaging IPs in TB care?**
11. **In your opinion, what could be challenges for engagement?**

    Probes:

- Coordination
- Communication
- Power relations
- Quality of care
- Support
- Discrimination
- Policy

- Resources
- IPs commitment

12. **Would you be willing to work with IPs if engaged by the government? OR, how do you feel about working with IPs in TB care?**
13. **Do you think the health workers like doctors, ASHAs would respond positively to IPs engagement in the formal TB program? Or do you see any challenges?**
14. **Are you aware of any government plans to engage IPs formally in TB program?**
15. **In your opinion, how could government facilitate IPs engagement in TB care? Training – Ask for details: What kind, duration, and specific content to cover?**

    Probes:

    Recognition and appreciation - Ask for more details

    Support – Ask for more details

    Incentive or remuneration – Ask for more details

    Policies and guidelines – Ask for more details

    System like referrals – Ask for more details
16. **What should be the immediate next step to engage IPs in TB care?**
17. **The state government recognizes IPs in West Bengal. Do you think the recognition has changed IPs engagement or relationship with the formal system?**
18. **Is there any other information that you would like to share with me?**

Thank you for your participation!!!
