# Supplementary material for: Barriers and facilitators to informal healthcare provider engagement in the national tuberculosis elimination program of India: An exploratory study from West Bengal
Source: PLOS Glob Public Health. 2023 Oct 4;3(10):e0001390. doi: 10.1371/journal.pgph.0001390 (PMC10550149; doi:10.1371/journal.pgph.0001390)
Supplement: S3 File — (PDF) [file pgph.0001390.s003.pdf]

Title: Barriers and facilitators to informal healthcare provider engagement in the national tuberculosis program of India: an exploratory study from West Bengal

Barriers:

S. N	Codes [Phase 2]	Merging of codes	S. N	Sub-themes [Phase 3 and 4]	S. N	Broader themes [Phase 5]
1	Difference in an understanding of engagement between the formal and informal sector		1	Difference in an understanding of “integration” between two system	1	*Non-alignment between groups regarding “integration” expectations*
2	Existing unclarity regarding the role of IPs in TB care		2	Unclarity in professional role and identity of IPs in TB care	2	*Ambiguity in role of IPs in TB care*
3	Lack of clear policy or guidance on IPs engagement					
4	Concerns pertinent to community-level formal health workers like ASHAs					
5	IPs' availability due to their clinical practice hours		3	IPs function as a care provider in the community<br>+ (Merged)<br>Reported low level of TB care knowledge among IPs	3	*Issues pertinent to IPs role as care provider*
6	Community concerns about IPs					
7	Lack of systematic monitoring system		4	Lack of streamlined monitoring and reporting system for IPs in TB care	4	*Lack of structured tracking and monitoring system*
8	Non-integration of IPs in current TB reporting system					
9	Low participation of IPs in current engagement		5	Gap in the current engagement program	5	*Observed gap in current engagement with IPs*
10	Low performance of IPs in current engagement					
11	Power dynamics between the formal and informal system		6	Existing power functions between the formal and informal system	6	*IPs acceptance and prevailing distrust between stakeholders in two system*
12	Reported low level of TB care knowledge among IPs		7	Reported low level of TB care knowledge among IPs		Merged

Facilitators:

S. N	Codes [Phase 2]	Merging of codes	S.N	Sub-themes [Phase 3 &4]	S.N	Broader themes [Phase 5]
1	IPs personal commitment towards their community		1	Social connection shared by IPs with their community<br><br>+<br><br>Convenient services offered by IPs	1	*Accessible and socially accepted healthcare delivered by IPs*
2	Good knowledge of the community they serve					
3	Good relationship with the community they serve					
4	Existing engagement of IPs in TB care		2	Flourishing recognition and engagement of IPs with the formal system	2	*Evolving recognition and engagement of IPs in formal health system*
5	Existing good relationship of IPs with the formal system					
6	Existing informal collaboration between health system and IPs					
7	Recognition of IPs importance by formal health providers					
8	Existing incentive system in TB program		3	Incentive system in National TB Program	3	*Inclusive incentive system in current National TB Program*
9	Convenient services offered by IPs		4	IPs as preferred providers in rural/underserved areas		Merged
10	IPs providing care in underserved areas					Merged
11	IPs role as primary care providers		5	Role of IPs as a primary care provider<br>+<br>IPs providing care in underserved areas	4	*IPs as primary care provider in rural and underserved areas*
12	Willingness among IPs to work with formal system		6	Optimism expressed by both groups toward working together	5	*Willingness expressed by both groups towards working together*
13	Willingness in the formal system to engage IPs					

Phase 6: Final overarching themes

S. N	Final themes	Sub-themes	Category
1	IPs position and capacity as a care provider	Accessible and acceptable healthcare services	
		IPs role as a primary care provider	
		Competing tasks and priorities	
		Quality of care	
2	Policy and system-level drivers and prohibitors	Lack of role clarity in TB care	
		Lack of systematic monitoring system	
		Inclusive incentive system	
		Evolving recognition in health system	
		Gaps in current engagement program	
3	Relationship between informal and formal systems	Willingness to work together	
		Expressed distrust between stakeholders in two systems	

Color legend:

Barrier

Facilitators
