# Supplementary material for: Barriers and facilitators to informal healthcare provider engagement in the national tuberculosis elimination program of India: An exploratory study from West Bengal
Source: PLOS Glob Public Health. 2023 Oct 4;3(10):e0001390. doi: 10.1371/journal.pgph.0001390 (PMC10550149; doi:10.1371/journal.pgph.0001390)
Supplement: Attachment — Submitted filename: Response to reviewers.docx [file pgph.0001390.s004.docx]

24 July 2023

The Editor,

PLOS Global Public Health

Dear Editor,

Re: Barriers and facilitators to informal healthcare provider engagement in the national tuberculosis program of India: an exploratory study from West Bengal

We thank the two external reviewers and you for the valuable comments that have allowed us to strengthen our manuscript. We have carefully reviewed the manuscript to address the reviewers' comments. Below, we provide a detailed response to the comments (highlighted in red are the changes made in the revised manuscript based on the reviewers’ comments and highlighted in blue are the additional response to the comments). We have also provided a tracked changed version of the manuscript highlighting the changes made to the originally submitted version.

**Comments from the editor:**

1. In the ethics statement in the Methods, you specified that verbal consent was obtained. Please provide additional details regarding how this consent was documented and witnessed, and state whether this was approved by the IRB.

**Response:** Thank you for your comment. More detail has been added to the manuscript as follows:

For all participants, informed verbal consent was taken, an approach approved by both ethics’ committees. Before commencing data collection, the research assistants (RAs) presented the verbal consent script and allowed participants to ask any questions. Once participants indicated their understanding of the research, their consent for the study was audio-recorded. These recordings were collected and stored separately from the study data to maintain confidentiality.

Please see line 199 on page 7 in the revised manuscript.

2. In the online submission form, you indicated that "The data underlying this article will be shared on reasonable request to the corresponding author". All PLOS journals now require all data underlying the findings described in their manuscript to be freely available to other researchers, 1. In a public repository, 2. Within the manuscript itself, or 3. Uploaded as supplementary information.

**Response:** We apologize for any confusion caused by our response in the initial submission. The manuscript contains all the necessary information regarding the study findings. Therefore, we kindly request to revise our response to the second option, "within the manuscript itself."

**Reviewer 1:**

1) This paper fills an important gap by providing insight into engagement of informal providers with the National TB Program (NTP) in India.

**Response:** We thank the reviewer for appreciating our work.

2) A major limitation of this work is limiting the study participants to health providers. As stated, the objective was to “explore factors that influence the engagement of IPs in India’s NTEP”. Engagement of informal providers in the NTEP is has a policy connotation, as the study also identifies (Table 2, major theme, “Policy and system-level drivers and prohibitors”). However, there was no involvement of NTEP managers, policymakers, or health department who either design or implement provider engagement policy of the NTEP, for example, state TB officer. This limits the scope of findings because not all the barriers (or facilitators) may have been captured in this analysis. While it doesn’t necessarily negate the findings in this study, it certainly limits the ability to offer potential pathways of building IP engagement. This should be clearly laid out as a limitation of this work.

**Response:** We appreciate the reviewer's valuable comment. While we made an effort to include formal healthcare providers up to the state level, it was not feasible to include providers at the central level due to the challenges posed by the ongoing COVID-19 pandemic during the data collection period. However, we acknowledge the importance of their involvement in the National TB Program of India, as highlighted by the reviewer. In response to this feedback, we have included this as a limitation in the manuscript as follows:

Third, the study was limited to the views of providers up to the state level. The central level TB officials involved in policy and guidelines were not involved due to the difficulties in access during the ongoing COVID-19 pandemic at the time of data collection.

Please see line 598 on page 19 in the revised manuscript.

2) The term informal health providers should be clearly defined. This is particularly important because the terminology can be interpreted differently depending on context and geographical location. This should be clearly distinguished from other terms used in the paper: formal health provider (FP), untrained allopathic practitioners (UAP). Consider explaining how this relates to traditional healers and AYUSH practitioners.

**Response**: Thank you for bringing attention to this important aspect of the paper. We have taken your feedback into consideration and included an additional section in the revised manuscript to provide clarity on the usage of the term.

The term "IPs" encompasses a diverse range of providers, including traditional health practitioners, traditional birth attendants, drug sellers, chemists, and untrained allopathic practitioners [UAPs]. These providers typically operate outside the formal healthcare system and often lack the necessary certification for the services they offer in the community [5-7]. In this study, we specifically enrolled UAPs whose primary practice is based on the allopathic system of medicine [8]. Therefore, moving forward, the term "IPs" in this paper specifically refers to UAPs.

Please see line 66 on page 3 in the revised manuscript.

3) [Abstract]

I suggest revising abstract to improve readability. Specifically,

1. the introduction can be shortened by focusing on informal providers’ lack of engagement with NTP

2. bullets can be turned into paragraph

3. removing line 29-30, “Two members of the research …”, “This is the first study …”

4. Adding detail on Braun and Clark framework’s theoretical underpinning – thematic analysis

5. Adding detail on participants in the study

6. Perhaps, a conclusion that goes beyond repeating that barriers need to be addressed. If not, consider removing the conclusion.

**Response:** Thank you for your helpful feedback. We have revised the abstract based on your feedback in the updated version.

Please refer to the abstract in the revised manuscript.

4) [Introduction]

I suggest moving lines 96-103 to methods and results, as appropriate. It explains the scoping review that was undertaken to inform the present study. The authors can also consider moving these details to supplementary file S1.

**Response:** Thank you for your suggestion. We have taken it into consideration and made significant reductions in the section. Additionally, we have relocated the majority of its content to supplementary file 1, as suggested. In the supplementary file, readers will find a comprehensive description of the rapid review conducted to inform our study.

Please refer to supplementary file S1.

5) [Methods]
1. Line 130: suggest using constructivism as an epistemology and not paradigm.

Response: We appreciate your advice regarding the use of terminology. we made the following revision:

The study is guided by the epistemology of constructivism. At the core of this orientation is the recognition that knowledge is socially constructed, and researchers actively engage in comprehending experiences from the participant's viewpoint [42].

Please see line 130 on page 5 in the revised manuscript.

2. Line 178-190: add details on transcription, translation, and language used for analysis. Consider adding how the analysed outputs in English were related back to the original data in vernacular.

**Response:** Thank you for your valuable comment regarding the details on transcription, translation, and language used for analysis. We have included the following information.

The interview transcripts were transcribed by a research assistant (RA) who was actively involved in the data collection process. Given that the RA had high-level skills in both languages, all interviews were directly transcribed into English. To ensure the accuracy and quality of the translations, a subset of the transcripts was independently verified by a team member from the Liver Foundation who is proficient in both Bengali and English. The verified transcripts in English were then discussed with the co-author (KB) to ensure consistency and maintain the integrity of the data during the analysis phase.

Please see line 215 on page 7 in the revised manuscript.

**Reviewer 2:**

1) The study which explores the barriers and facilitators to informal healthcare provider engagement in the national tuberculosis program in a district of West Bengal in India is relevant. Congratulations to the authors for a good study! It has also been written in an excellent way!

**Response:** We thank the reviewer for appreciating our work.

2) Abstract: the authors wrote that “This is the first study that has consulted IPs and FPs to explore factors influencing IPs’ engagement in India’s NTEP”- As the study is done in an ‘ideal’ setting in one of the districts in India, request not to generalise it to ‘India’s NTEP’.

**Response:** Thank you for your feedback. We have revised the abstract to reflect your suggestions.

Please refer to the abstract in the revised manuscript.

2) When the authors mentioned about FP (formal health care) providers, a reader will be under the impression that those are formal private providers. The impression was created by the authors in introduction section talking about ‘formal’ and ‘informal’ private providers. I do not know if there are ‘informal’ public providers. The FPs mentioned throughout in this paper are NTEP State and District Program Managers and ASHAs. Instead of calling them as ‘FP’ in this paper, it would have been better to call them as NTEP state and district program managers and ASHAs. Kindly consider so as to avoid confusions.

**Response:** Thank you for highlighting this important issue. To mitigate this confusion and provide clarity, we have incorporated a definition of FPs in the revised version of the paper. By utilizing the acronym FP throughout the document, we aim to streamline the terminology and facilitate easier reference to these providers. We believe that including the following definition will enhance the understanding of readers in line with your helpful suggestion.

Please note that the Formal Providers (FPs) who participated in this study were affiliated or engaged with the National Tuberculosis Elimination Program (NTEP) of India, including personnel at the state and district levels responsible for policy and program implementation, as well as Accredited Social Health Activist (ASHAs) involved in the delivery of TB care services at the community level. Henceforth, the term FPs will be used to indicate these providers who are affiliated or engaged with the NTEP.

Please see line 155 on page 5 in the revised manuscript.

3) Similarly, authors used ‘Formal’ TB Program in multiple places. It gives an impression that there is informal TB program also. I suggest to rename it as National TB Program.

**Response:** We appreciate your helpful suggestion. We have made changes throughout the manuscript, using either, “National TB program” or “National TB Elimination Program” as appropriate.

4) There is a major confusion felt by the reader which was either in the minds of IPs (or in the mind of authors). In introduction IPs were described as untrained allopathic practitioners and their counterparts at formal system was mentioned as Formal Allopathic Practitioners (formal private sector). While moving down the manuscript, IPs were having issues with considering them as community volunteers, and they were having issues with ASHAs. Responses from IPs were like they need compensation to work outside usual hours (as a community volunteer?) and some of them were ready to take up full time job for NTEP (as what?). Authors were hoping to offer them sharing the responsibilities of ASHAs in results and discussions. [“IPs could also support ASHAs by undertaking some of the TB care roles assigned to them, mainly as both groups of providers’ function at the community level, and ASHAs are reported to be overburdened with multiple service delivery responsibilities”]. Request the authors to try and see if there is a way to clarify these entire confusions or at least tell the readers whose confusion was that.

Response: Thank you for your insightful comment. We have taken your suggestions into consideration and made significant revisions to the manuscript, particularly in the results, discussion, and conclusion sections, to address the concerns you raised. Please refer to the revised version of the manuscript.

Regarding the issues with ASHAs, we noted that the observed challenge stems from the power dynamics between the formal and informal healthcare systems. Our findings indicate that IPs perceive themselves as independent care providers and emphasise the importance of financial compensation for their services within the NTEP. They consider working as an IP as their primary occupation and rely on it for their livelihood. Therefore, their willingness to engage in the NTEP is contingent upon receiving appropriate remuneration for their contributions rather than working as volunteers.

In our discussion, we have discussed the potential for IPs to assume some of the roles currently assigned to ASHAs, citing a recent study that found comparable treatment outcomes between ASHAs and IPs. However, the specific mechanisms for effectively engaging IPs in these roles require further exploration, which falls outside the scope of this study. We have a second paper currently under review, which explores the perspectives of IPs and formal providers regarding the current and potential roles of IPs in TB care. This forthcoming paper will provide comprehensive insights into how IPs envision their involvement in different aspects of TB care.

5) Among the IPs- 10 were engaged and 13 were not engaged. A reader would love to know the reasons for non-engagement, in a place where a positive policy existed, since they constituted the majority. Kindly consider providing more insights to it.

**Response:** Thank you for your suggestion. We have added the following information in the revised version.

The West Bengal state government’s efforts to engage IPs for the referral of presumptive TB cases faced several challenges. The first noted challenge was the low coverage, as only a limited number of IPs participated in the program. As one IP shared, “Some IPs from my area went for the training at BPHC, but I don’t think all of them are currently referring patients” (IP, 54 M, High Edu.).

One IP who was not engaged in the program expressed dissatisfaction with being excluded and mentioned that neither they nor other IPs in the neighbourhood were invited to participate. This exclusion could be attributed to the enrolment process, as the listed IPs were only designated the role of referring TB patients. A formal provider stated, "We asked to submit the block-wise list of practicing IPs. Once we enlisted their name, they were assigned the role to formally refer presumptive TB patients after undergoing training. (FP)" One engaged IP suggested that involving local IP associations could enhance the participation of all IPs in the program.

Please see line 448 on page 15 in the revised manuscript.

6) The entire thoughts on “Competing tasks and priorities?” was a bit unclear. The only task assigned to IPs was to screen and refer a presumptive TB for testing? How can the “competing tasks and priorities” affect that?

**Response:** Thank you. We agree that the previous version was unclear, so we have made revisions to the section as follows.

Despite their wide acceptance and close proximity to the community, engaging IPs in any formal programs, including TB care requires careful consideration. They reported that working as an IP was their primary occupation and sole means of livelihood, so some hesitancy was noted among some IPs to dedicate substantial time to the National TB care program. One IP expressed this concern, stating, “This is my primary profession, so if I work in the National TB Program, it will impact my clinic hours and patient load. Therefore, it is important to receive financial support for our work in TB care” (IP, 38 M, Post-Sec.). As indicated in the quote, an agreement was noted among IPs to adjust their work schedule if the government provided some remuneration for their work in TB care. FPs also acknowledged this challenge, with one remarking, “They can refer presumptive TB cases from their clinic as part of their regular practice. However, if we restrict them to a fixed work schedule and assign responsibilities outside of their clinic, they may become demotivated as working as an IP is their primary occupation" (FP). This underscores the necessity of aligning the roles assigned to IPs with their existing work structure.

Please see line 332 on page 11 in the revised manuscript.

7) Similarly, “need for more incentives” and “compensate for work hours outside of their regular practice”. Formal private health sector is also being offered the same incentive [Informant Incentive or Notification Incentive] for the task. How is it justified to offer more incentive? Are there anything which can work other than financial incentives? Any thoughts?

**Response**: Thank you for your comment. In our manuscript, we have discussed the current gaps in the incentive system, particularly the loopholes that affect the effectiveness of incentives for IPs. We emphasize the importance of addressing these gaps to ensure that IPs receive appropriate incentives and that their engagement in the NTEP is sustainable. Furthermore, we have highlighted the desire expressed by formal providers to assign additional roles to IPs in the NTEP, such as treatment supporters. This suggests there is potential to expand the scope of responsibilities for IPs within the program. Regarding compensation for work outside their regular practice, our original thinking was to address the additional tasks assigned to IPs, such as contact tracing. We agree that the compensation should align with the norms of the NTEP and not exceed the benefits provided to formal providers.

In the discussion, we have emphasized the importance of training and government recognition to enhance the engagement of IPs in TB care. However, considering that working as an IP is their primary occupation, it is crucial to provide appropriate compensation to recognize their contributions to the NTEP.

8) The reader could see so many issues with non-receipt of incentives from the results presented, due to multiple reasons. Still do we need to promote more financial incentives?

**Response:** We acknowledge your point that incentives are not the sole motivating factor for IPs' engagement in the NTEP. However, we believe it is crucial to provide incentives to IPs on par with formal providers to establish a fair system and ensure the sustainable engagement of IPs. In our paper, we emphasize the issue of non-receipt of incentives, as it was commonly reported by currently engaged IPs and is an important issue that needs to be addressed to strengthen the engagement of IPs in the NTEP.

9) Author states that “Practising as an IP is illegal and can attract punishment in India”. The author also states that “NTEP need to formulate guidelines for IPs” – how can NTEP formalise an illegal practise? Any thoughts on this?

**Response:** The inclusion of the informal sector (such as IPs) in TB care has been emphasized in global TB policies and guidelines, such as the End TB Strategy and WHO guidance on private sector engagement. These documents recommend developing tailored engagement strategies based on the context. Given the widespread presence of IPs in India's health system and their role as the first point of contact for many TB patients, the engagement of IPs in the NTEP cannot be overlooked. While we are unable to comment on IPs' engagement from a systemic perspective, our recommendations are based on two key pieces of evidence. Firstly, the Government of West Bengal's initiative to recognize and train IPs, as discussed in our manuscript, is a novel approach that has expanded to include IPs in other formal programs, including the COVID-19 response. Secondly, there is a growing body of evidence demonstrating the positive impact of IPs in strengthening TB care programs when appropriate training is provided. Therefore, we believe it is crucial to conduct further research on topics related to IPs and to address their inclusion in policies. Neglecting the existence of IPs in the health system will not address the underlying issues in the long run.

10) One of the suggestions of the authors is “One possible solution could be to integrate IPs in Nikshay, NTEP’s patient management system, which enables the monitoring of patients from the stage of presumptive identification to completion of treatment.”- there is already provision in Nikshay where any volunteer can enrol and refer presumptive TB patient by themselves. The person who referred can know the status and feedbacks. It is linked to informant incentive (Rs 500 INR per month) also. If this feature of Ni-kshay is utilised, most of the problems cited will be solved. Request author to reconsider the statements.

**Response:** Thank you for your feedback. We believe treating IPs as private providers rather than volunteers in TB care is crucial for their prioritization and active engagement in improving TB care outcomes. This aligns with the classification of IPs as private providers in global TB policies, and it emphasizes the need for the NTEP to develop appropriate strategies to engage with IPs similar to other formal private sector providers.

Regarding the referral system, we acknowledge the limitations of the current paper-based forms provided to IPs during training by the government. In this regard, our recommendation is to utilize existing digital tools such as Nikshay to address the challenges associated with the referral system. However, we have made no comment on the version of Nikshya, which can be utilized by IPs, as this was not explored in this study.

11) In the conclusion authors stated that “Considering the existing body of evidence highlighting the role, importance, and potential of IPs in TB care, strengthening their engagement in the NTEP is essential.”- Though the statement is correct, it can’t be the conclusion for this particular study which explored facilitators and barriers for engagement of IPs.

**Response:** We thank you for your valuable feedback. We have removed this sentence and also thoroughly revised the conclusion section in the revised version.

Please see line 607 on page 19 in the revised manuscript.

12) In methods, it was mentioned that the tool was tested among four participants. Were they part of 17/7? Are those results included in the analysis? Kindly clarify

**Response:** The pilot data were not included in the final analysis.

13) Given the current provisions with NTEP (Informant incentive, Training, Treatment Supporter incentives) and the legal restriction - what recommendations the authors can make to NTEP based on the findings?

**Response:** Thank you for your comment. We have included recommendation along with the conclusion in the revised version as follows.

In conclusion, this study provides valuable insights into the barriers and facilitators of engaging IPs in the NTEP. The findings highlight the vital role IPs play as primary care providers, serving as an initial point of contact for TB patients. To optimize their engagement, several recommendations arise. Firstly, national TB policies and guidelines must explicitly address IPs, clarifying their roles and responsibilities within the NTEP. This will establish a framework for their involvement and provide much-needed clarity. Secondly, targeted training programs should be developed to enhance IPs' knowledge and skills in TB care, focusing on areas such as accurate case identification and referral practices. These programs should address identified knowledge gaps and improve the quality of care delivered by IPs. Thirdly, the incentive system for IPs needs to be streamlined to compensate them for their efforts and sustain their engagement. This includes integrating IPs into the NTEP's patient management system to enable systematic patient tracking and addressing the limitations of the current paper-based referral system. Fourthly, creating a supportive environment is crucial to foster collaboration and address incidents of neglect and discrimination experienced by some IPs. Policies and guidelines should be implemented to promote an inclusive environment and encourage partnerships between IPs and FPs. Lastly, the recognition of IPs by the West Bengal government has shown positive outcomes, emphasizing the importance of formal acknowledgment at the health system level. Recognition and effective utilization of IPs can lead to improved access to quality care, enhanced early TB case detection, and ultimately contribute to the overall goal of TB elimination.

Please see line 607 on page 19 in the revised manuscript.
